# Response of the Urothelial Carcinoma Cell Lines to Cisplatin

**DOI:** 10.3390/ijms232012488

**Published:** 2022-10-18

**Authors:** Andrea Holíčková, Jan Roška, Eveline Órásová, Vladimíra Bruderová, Patrik Palacka, Dana Jurkovičová, Miroslav Chovanec

**Affiliations:** 1 Biomedical Research Center, Department of Genetics, Cancer Research Institute, Dúbravská cesta 9, 845 05 Bratislava, Slovakia; 2 Department of Genetics, Faculty of Natural Sciences, Comenius University, Mlynská Dolina, Ilkovičova 6, 842 15 Bratislava, Slovakia; 3 National Cancer Institute, Klenová 1, 833 10 Bratislava, Slovakia; 4 2nd Department of Oncology, Faculty of Medicine, Comenius University in Bratislava, Klenová 1, 833 10 Bratislava, Slovakia

**Keywords:** bladder cancer, cisplatin, DNA damage repair and tolerance, nucleotide excision repair, homologous recombination, translesion DNA synthesis

## Abstract

Cisplatin (CDDP)-based chemotherapy is the standard of care in patients with muscle-invasive bladder cancer. However, in a large number of cases, the disease becomes resistant or does not respond to CDDP, and thus progresses and disseminates. In such cases, prognosis of patients is very poor. CDDP manifests its cytotoxic effects mainly through DNA damage induction. Hence, response to CDDP is mainly dependent on DNA damage repair and tolerance mechanisms. Herein, we have examined CDDP response in a panel of the urothelial carcinoma cell (UCC) lines. We characterized these cell lines with regard to viability after CDDP treatment, as well as kinetics of induction and repair of CDDP-induced DNA damage. We demonstrate that repair of CDDP-induced DNA lesions correlates, at least to some extent, with CDDP sensitivity. Furthermore, we monitored expression of the key genes involved in selected DNA repair and tolerance mechanisms, nucleotide excision repair, homologous recombination and translesion DNA synthesis, and show that it differs in the UCC lines and positively correlates with CDDP resistance. Our data indicate that CDDP response in the UCC lines is dependent on DNA damage repair and tolerance factors, which may, therefore, represent valuable therapeutic targets in this malignancy.

## 1. Introduction

Bladder cancer (BC) is the 10th most common cancer in the world, and its incidence is steadily rising worldwide [1]. Urothelial carcinoma covers almost 90% of all BCs [2]. Although 70% of BC patients have a non-muscle-invasive cancer (NMIBC) at time of diagnosis, they relapse and progress to muscle-infiltrating bladder cancer (MIBC) in approximately 1 in 5 cases [3]. Patients with MIBC account for about 30% of all cases, in which a radical cystectomy (RC) represents the gold standard of treatment [4]. From 40% to 67% of patients with pT3-T4a or lymph node-positive disease relapse after RC alone, with a poor 5 year overall survival (OS) of 25–30% [5,6]. Cisplatin (CDDP)-based neoadjuvant chemotherapy (NAC) is associated with a survival benefit and an absolute increase in 5 year survival of 8% [7]. Similarly, CDDP is a backbone of regimes used in the first line setting of inoperable locally advanced or metastatic disease [4]. However, up to 50% of BC patients do not respond to CDDP-based chemotherapy in terms of pathologic complete response after NAC [8] and objective response on computed tomography scans during and after treatment of an advanced disease [9]. These data represent a grave problem in daily practice for both the patients and their clinical oncologists.

The main molecular target of CDDP is DNA, where it forms monoadducts, intra- (IaCLs) and inter-strand (ICLs) cross-links, and DNA-protein cross-links. Repair of CDDP-induced DNA damage is a complex process and involves coordinated action of nucleotide excision repair (NER), homologous recombination (HR) and translesion DNA synthesis (TLS) [10]. NER is a versatile DNA repair system that, in addition to CDDP-induced DNA damage, also removes a wide range of bulky DNA damage. This damage deforms the DNA structure, blocks replication and induces apoptosis [11]. NER involves at least 30 proteins that physically interact with each other at different stages of the repair process. The first steps of NER involve the recognition and verification of DNA damage by the XPA protein. This protein further contributes to the coordination of the assembly of downstream NER complexes [12,13]. HR is crucial for the repair of severe DNA damage such as DNA double-strand breaks. Genes from the *RAD51* family, *RAD51* and five *RAD51*-related genes (*XRCC2*, *XRCC3*, *RAD51B*, *RAD51C* and *RAD51D*), have critical and non-redundant functions in this pathway, and their loss is embryonically lethal. RAD51 is a central HR protein catalysing DNA strand transfer between a broken sequence and its undamaged homologous template to allow re-synthesis of the damaged region. This transfer causes the extrusion of the non-complementary intact strand, forming a “D-loop” that enlarges as DNA synthesis progresses across the break site. Finally, the enzymatic cleavage of the cross structure occurs, the so-called Holliday junction (reviewed in [14,15,16]). TLS has a special role in the elimination of the cytotoxic damage induced by CDDP. In contrast to replicative DNA polymerases, TLS DNA polymerases exhibit a low fidelity in synthesizing an intact DNA template. Their primary role is to protect cells from the fatal consequences of DNA damage by bypassing it through the multiprotein complex. The main components of this complex are DNA polymerases from the Y family (Pol η, Pol ι and Pol κ encoded by the *POLH*, *POLI* and *DINB1* genes), which carry out the function of “inserters”, polymerase ζ (consisting of the REV3L catalytic and REV7 regulatory subunits) from family B, which functions as an “extender”, and the REV1 protein, which acts as an interaction partner between PCNA and other DNA polymerases performing coordination and regulatory functions (for a review, see [17,18]).

Deregulated NER, HR or TLS factors can increase the risk of malignant transformation and potentially lead to chromosomal rearrangements, induction of mutations and aberrant DNA repair [19,20]. Aberrant DNA repair along with DNA damage tolerance represent a potential avoidant strategy to the cytotoxic effects of CDDP in tumour cells, and, therefore, they are considered as the main mechanisms contributing to CDDP resistance [21]. Indeed, several studies have already confirmed the clinical relevance of some DNA repair factors, and several of them are currently the subject of targeted clinical trials [22,23,24,25]. The present work was aimed at characterization of a panel of the urothelial carcinoma cell (UCC) lines in terms of their CDDP response. Hence, viability and the kinetics of DNA damage induction and repair have comprehensively been examined in these cell lines after CDDP treatment. In addition, to reveal potential DNA damage repair and tolerance biomarkers associated with CDDP response, the gene expression of selected NER, HR and TLS factors before and after CDDP exposure has been quantified and correlated with viability in the UCC lines.

## 2. Results

### 2.1. Viability of the UCC Lines after CDDP Treatment

**Figure 1:**
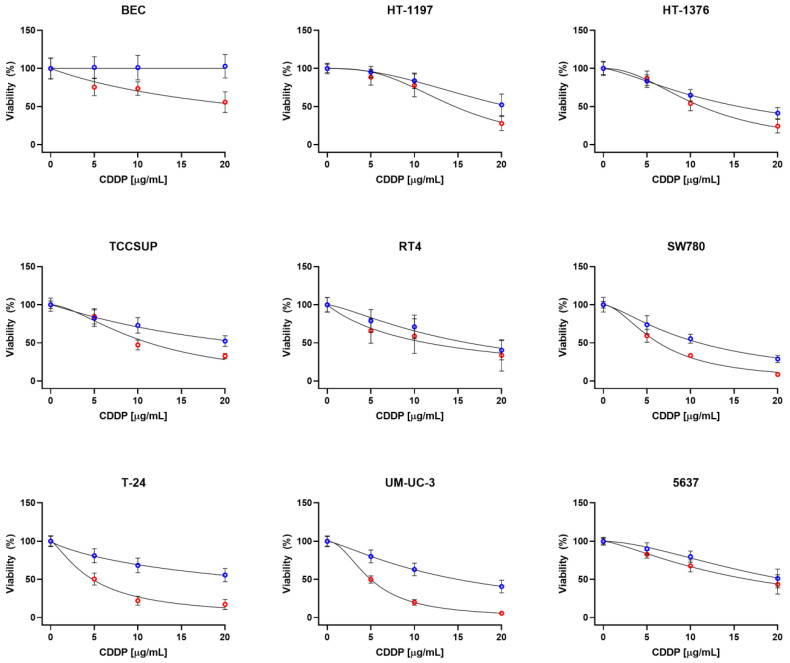
Viability of the UCC lines and BEC after 2 h CDDP exposure followed by 24 (blue) and 48 (red) h post-incubation under standard growth conditions. Values are shown as the mean ± SD (*n* = 8 of three biological replicates).

Firstly, we have examined viability of the UCC lines after CDDP treatment. Primary culture of healthy bladder epithelial cells (BEC) was included for comparison. As mentioned in Material and Methods, four different CDDP treatment conditions were used: 2 h CDDP exposure followed by 24 or 48 h post-incubation period under standard growth conditions and continuous 24 or 48 h CDDP exposure. As evident, there is substantial difference in the CDDP sensitivity among the UCC lines that is strongly dependent on treatment conditions. In case of 2 h exposure and 24 h post-incubation, the HT-1197, TCCSUP, 5637 and T-24 cell lines could be classified as resistant, while SW780 as sensitive. The rest of cell lines displayed intermediate/moderate CDDP sensitivity. This pattern slightly differs for 48 h post-incubation: while the HT-1197 and 5637 can still be considered as resistant, the SW780 as sensitive, and the HT-1376 and RT4 as intermediately/moderately sensitive cell lines, TCCSUP, T-24 and UM-UC-3 lost their resistant and intermediate/moderate phenotype and became CDDP sensitive. Surprisingly, BEC appears to be the most resistant cell line under these CDDP treatment conditions (Figure 1 and Table 1).

**Figure 2:**
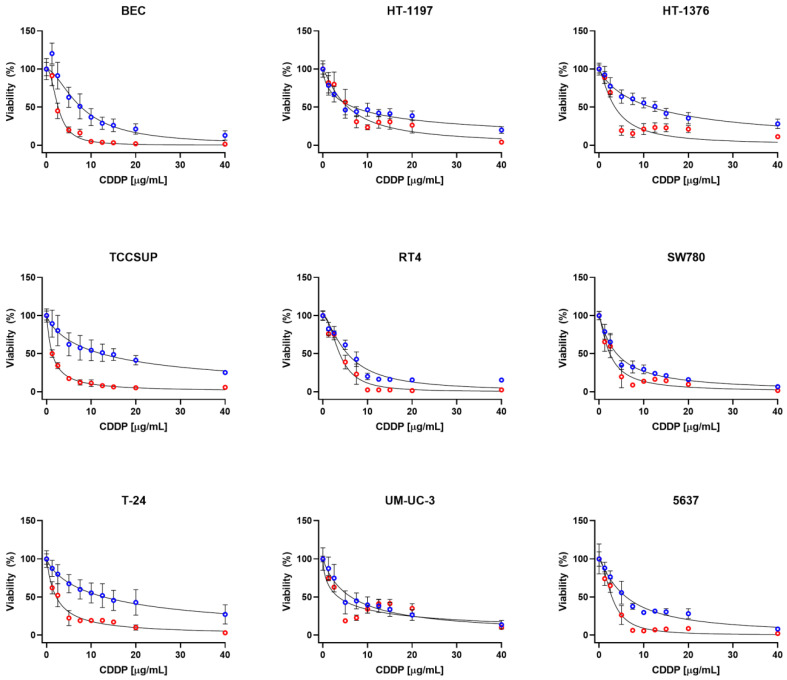
Viability of the UCC lines and BEC after continuous 24 (blue) and 48 (red) h CDDP exposure. Values are shown as the mean ± SD (*n* = 8 of three biological replicates).

In case of continuous 24 h treatment, the UCC lines HT-1376, TCCSUP and T-24 display CDDP resistant phenotype, while SW780 can be considered as sensitive. The rest shows intermediate/moderate sensitivity to CDDP. Significantly different pattern of CDDP sensitivity was revealed after continuous 48 h treatment, where the HT-1197, HT-1376, RT4 and UM-UC-3 cell lines were CDDP resistant, 5637, SW780 and T-24 intermediately/moderately sensitive, and TCCSUP sensitive to CDDP (Figure 2 and Table 1). In this case, BEC showed rather intermediate/moderate phenotype after CDDP treatment. 

In summary, all above data point out to the fact that viability of the UCC lines after CDDP exposure is strongly dependent on treatment conditions, particularly on the time of treatment and post-incubation recovery.

### 2.2. Kinetics of DNA Damage Induction and Repair in the UCC Lines after CDDP Treatment

Next, we monitored the kinetics of DNA damage induction and repair at 0, 4, 24 and 48 h after CDDP exposure by the SO-modified comet assay, also called a reverse comet assay. SO is an electrophile interacting with DNA bases at numerous sites (including the N7 position of guanine) leading to the formation of diverse DNA adducts [26,27,28,29,30], which represent alkaline labile sites and can be converted into DNA single-strand breaks [31], causing DNA to migrate into the tail of comet in alkaline comet assay. The platinum atom in CDDP binds primarily to the N7 position of purines, resulting in the formation of DNA monoadducts, IaCLs and ICLs [32], with ICLs being responsible for retaining most DNA in the head of comet in the classical comet assay. However, in the SO-modified assay, ICLs can be detected. The SO-modified comet assay can also likely detect CDDP-induced DNA monoadducts because of the competitive binding of SO and CDDP to the same DNA sites, leading to abolition of the SO effect by the presence of CDDP-induced DNA monoadducts.

**Figure 3:**
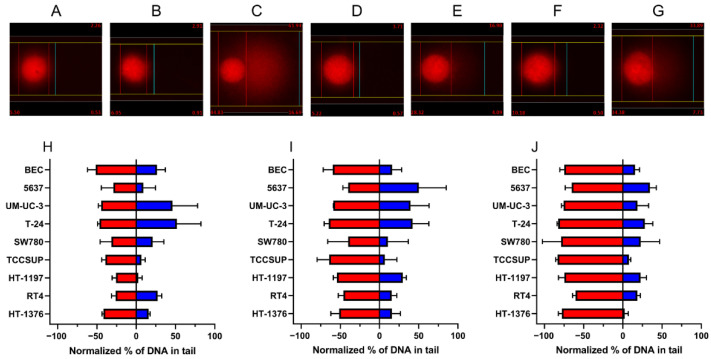
Representative images from the reverse comet assay and comparison of the DNA damage induction and repair in the UCC lines and BEC after CDDP treatment. Representative image for the RT4 cell line is shown. Untreated control (2.26% DNA in tail) (**A**), sample treated with 10 μg/mL CDDP (2.91% DNA in tail) (**B**), sample treated with SO (61.94% DNA in tail) (**C**), sample treated with 10 μg/mL CDDP at 24 h (3.71% DNA in tail) (**D**), sample treated with SO 24 h after 10 μg/mL CDDP treatment (16.90% DNA in tail) (**E**), sample treated with 10 μg/mL CDDP at 48 h (2.32% DNA in tail) (**F**) and sample treated with SO 48 h after 10 μg/mL CDDP treatment (33.89% DNA in tail) (**G**). Comparison of DNA induction and repair after 5 μg/mL CDDP treatment in BEC, 5637, UM-UC-3, T-24, and HT-1376 at 4 h, in TCCSUP at 0 h, and SW780, HT-1197 and RT4 at 24 h (**H**); after 10 μg/mL CDDP treatment in BEC, 5637, UM-UC-3, T-24, SW780 and HT-1376 at 4 h, and TCCSUP, HT1197 and RT4 at 24 h (**I**) and after 20 μg/mL CDDP treatment in BEC, 5637, UM-UC-3, T-24 and SW780 at 4 h, and TCCSUP, HT-1197, RT4 and HT-1376 at 24 h (**J**). Red bars represent the highest level of CDDP-induced DNA damage, irrespective of post-incubation time when it was reached. Blue bars represent DNA repair levels observed at 48 h after CDDP treatment. Data are presented as mean ± SD (*n* = 2) from three biological replicates.

As expected, CDDP induces DNA damage in all UCC lines in a concentration-dependent manner. However, the level of DNA damage induction slightly varies among individual cell lines (Figure 3 and Supplementary Figure S1). At 5 μg/mL CDDP, the highest DNA damage induction was observed in the BEC, T-24 and UM-UC-3 cell lines, followed by HT-1376, TCCSUP, SW780 and 5637. The lowest levels of CDDP-induced DNA damage were found in RT4 and HT-1197. At 10 μg/mL CDDP, the highest DNA damage induction was revealed in the T-24, TCCSUP, BEC and UM-UC-3 cell lines, followed by HT-1197, HT-1376, RT4, 5637 and SW780. At 20 μg/mL CDDP, the range of DNA damage induction among the cell lines differed the least, with the highest DNA damage levels being induced in the T-24, TCCSUP, UM-UC-3, HT- 1197 and BEC cell lines, followed by 5637, and the smallest levels were found in RT4 (Figure 3 and Supplementary Figure S1).

As in case of DNA damage induction, the UCC lines also differed in the level of repair of CDDP-induced DNA damage 48 h after CDDP treatment. At a dose of 5 μg/mL, the order of ability of the UCC lines to remove CDDP-induced DNA damage was as follows: T-24 ~ UM-UC-3 > RT4 ~ BEC > SW780 ~ HT-1376 > 5637 > TCCSUP > HT-1197 ~ virtually no DNA repair activity. After 10 μg/mL CDDP treatment, this ability was in order: 5637 > T-24 ~ UM-UC-3 > HT-1197 > HT-1376 ~ RT4 ~ BEC > SW780 > TCCSUP. Finally, DNA damage induced by 20 μg/mL CDDP treatment was repaired in the UCC lines in the following order: 5637 > T-24 > SW780 ~ HT-1197 > RT4 ~ BEC ~ UM-UC-3 > TCCSUP > HT-1376 ~ virtually no DNA repair activity. Collectively, DNA repair data indicate that a panel of the UCC lines used herein is endowed with differently efficient capacities to repair CDDP-induced DNA damage.

### 2.3. Relative Basal mRNA Expression of the DNA Damage Repair and Tolerance Factors

#### 2.3.1. Relative Basal mRNA Expression of the Nucleotide Excision Repair Factors

**Figure 4:**
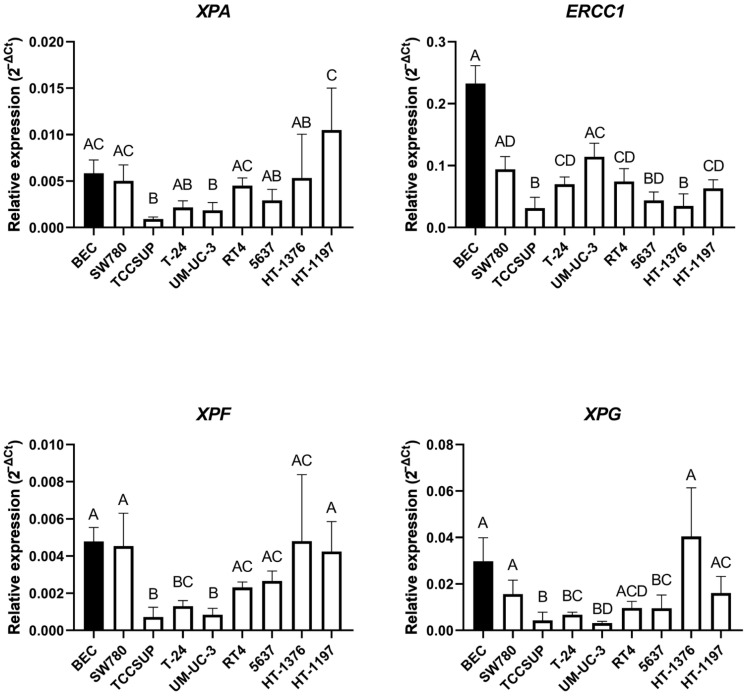
Relative basal gene expression of the selected NER factors in the UCC lines and BEC. Relative mRNA expression of *XPA* (**upper panel left**), *ERCC1* (**upper panel right**), *XPF* (**lower panel left**) and *XPG* (**lower panel right**). Values are reported as the mean with a 95% confidence interval from three biological and three technical replicates. Columns marked with the same letters did not show a statistically significant difference, while columns marked with different letters indicate a statistically significant difference using one-way ANOVA with Tukey post-hoc test for multiple comparisons.

We determined relative basal mRNA expression of the four key NER factors, *XPA*, *ERCC1*, *XPF* and *XPG*, in the UCC lines and BEC. In case of *XPA*, the highest relative basal expression was detected in the HT-1197 cell line, while the lowest expression was found in TCCSUP. Relative basal expression of *XPA* mRNA was statistically significantly higher in BEC compared to TCCSUP and UM-UC-3 (Figure 4, upper panel left). Of all UCC lines, the UM-UC-3 cell line showed the highest relative basal expression of *ERCC1* mRNA, which was statistically significant when compared with TCCSUP, HT-1376 and 5637. The lowest relative basal expression was detected in TCCSUP, and proved to be significant when compared to BEC, UM-UC-3, SW780, RT4, T-24 and HT-1197. Interestingly, the highest *ERCC1* mRNA basal expression was found in BEC and was statistically significantly higher compared to most of the UCC lines (Figure 4, upper panel right). Relative basal expression of *XPF* mRNA was highest in the HT-1376 cell line, with a significant difference compared to TCCSUP and UM-UC-3. The lowest basal level of *XPF* mRNA was expressed in the TCCSUP cell line, which was significantly lower compared to BEC, HT-1376, SW780, HT-1197, 5637 and RT4. Relative basal expression of *XPF* mRNA was higher in BEC compared to TCCSUP, UM-UC-3 and T-24 (Figure 4, lower panel left). Relative basal expression of *XPG* mRNA was highest in the HT-1376 cell line with a significant difference compared to UM-UC-3, TCCSUP, T-24 and 5637. The lowest basal expression of *XPG* was found in UM-UC-3, which was statistically lower compared to BEC, HT-1197, HT-1376, SW780 and RT4. BEC had higher basal expression of *XPG* mRNA compared to UM-UC-3, TCCSUP, T-24 and 5637 (Figure 4, lower panel right).

#### 2.3.2. Relative Basal mRNA Expression of the Homologous Recombination Factors

**Figure 5:**
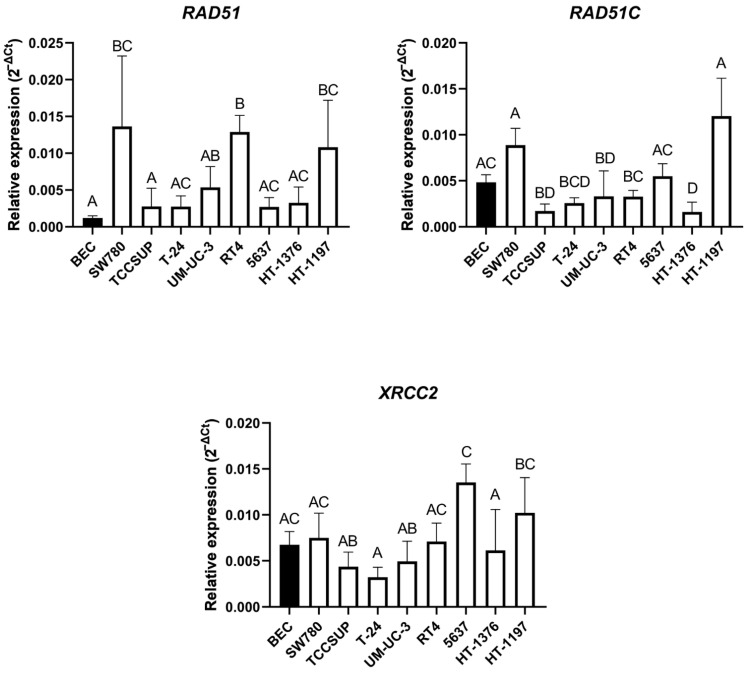
Relative basal gene expression of the selected HR factors in the UCC lines and BEC. Relative mRNA expression levels of *RAD51* (**upper panel left**), *RAD51C* (**upper panel right**) and *XRCC2* (**lower panel**). Values are reported as the mean with a 95% confidence interval from three biological and three technical replicates. Columns marked with the same letters did not show a statistically significant difference, while columns marked with different letters indicated a statistically significant difference using one-way ANOVA with Tukey post-hoc test for multiple comparison.

We also examined relative basal mRNA expression of the three HR factors, *RAD51*, *RAD51C* and *XRCC2*. The highest *RAD51* mRNA basal expression was observed in the SW780 cell line, in which the level of *RAD51* mRNA was significantly increased compared to BEC and TCCSUP. The TCCSUP, T-24, 5637 and HT-1376 cell lines expressed this gene at a significantly lower level when individually pairwise compared to SW780, RT4 and HT-1197. The lowest *RAD51* mRNA basal expression was found in BEC, where expression of this gene was significantly lower compared to the SW780, RT4 and HT-1197 cell lines (Figure 5, upper panel left). The highest basal expression of *RAD51C* was shown in the HT-1197 cell line, with a significant difference compared to the HT-1376, TCCSUP, T-24, RT4 and UM-UC-3 cell lines. The lowest *RAD51C* basal expression was detected in the TCCSUP cell line, when compared to BEC, 5637, SW780 and HT-1197. BEC had significantly higher expression of this gene compared to the HT-1376, TCCSUP and UM-UC-3 cell lines (Figure 5, upper panel right). The highest basal expression of *XRCC2* was observed in the 5637 cell line, which expressed this gene to significantly higher level compared to the T-24, TCCSUP, UM-UC-3 and HT-1376 cell lines. T-24 showed the lowest level of *XRCC2* mRNA which proved to be significantly lower compared to the HT-1197 and 5637 cell lines. *XRCC2* basal expression in BEC did not show a significantly different level of expression against any UCC line (Figure 5, lower panel).

#### 2.3.3. Relative Basal mRNA Expression of the Translesion Synthesis Factors

**Figure 6:**
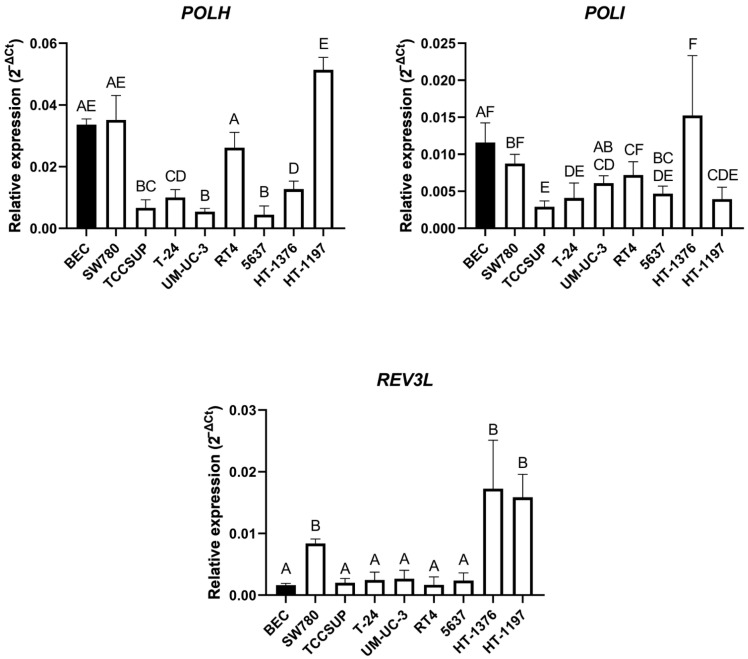
Relative basal expression of the selected TLS factors in the UCC lines and BEC. Relative mRNA expression of *POLH* (**upper panel left**), *POLI* (**upper panel right**) and *REV3L* (**lower panel**). Values are reported as the average with a 95% confidence interval from three biological and three technical replicates. Columns marked with the same letters did not show a statistically significant difference, while columns marked with different letters indicated a statistically significant difference using one-way ANOVA with Tukey post-hoc test for multiple comparison.

Finally, we measured relative basal expression of the three TLS factors, *REV3L*, *POLH* and *POLI*. The highest basal expression of *POLH* mRNA was displayed in the HT-1197 cell line, expressing this gene at a significantly higher level compared to the 5637, UM-UC-3, TCCSUP, T-24 and HT-1376 cell lines. The lowest expression of *POLH* was shown in the 5637 cell line and was statistically significant compared to the HT-1197, SW780, BEC, RT4, HT-1376 and T-24 cell lines. BEC had higher *POLH* expression compared to the 5637, UM-UC-3, TCCSUP, T-24 and HT-1376 cell lines (Figure 6, upper panel left). HT-1376 showed the highest *POLI* mRNA expression, with higher expression of this gene compared to TCCSUP, HT-1197, T-24, 5637 and UM-UC-3. The lowest level of *POLI* mRNA was observed in the TCCSUP cell line and this was lower compared to HT-1376, BEC, SW780, RT4 and UM-UC-3. BEC had a higher basal expression of this gene compared to the TCCSUP, T-24, HT-1197 and 5637 cell lines (Figure 6, upper panel right). The HT-1376, HT-1197 and SW780 cell lines showed significantly higher expression of the *REV3L* gene compared to other UCC lines and BEC (Figure 6, lower panel). 

#### 2.3.4. Correlation of Relative Basal Expression of Individual NER, HR and TLS Factors as Well as of the Cumulative Expression of the NER, HR and TLS Pathways with IC_50_ Values

**Figure 7:**
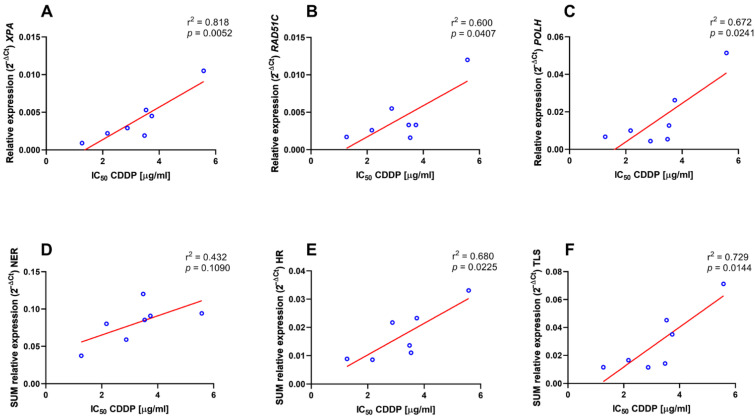
Correlation of relative basal mRNA expression of individual NER, HR and TLS factors as well as of cumulative expression of the NER, HR and TLS pathways with IC_50_ values of continuous 48 h CDDP treatment. Correlation of basal mRNA expression levels of *XPA* (**A**), *RAD51C* (**B**) and *POLH* (**C**), as well as of cumulative NER (*XPA* + *ERCC1* + *XPF* + *XPG*) (**D**), HR (*RAD51* + *RAD51C* + *XRCC2*) (**E**) and TLS (*REV3L* + *POLH* + *POLI*) (**F**) pathways with IC_50_ values. The respective values of the coefficient of determination (r^2^) and the *p* values are listed in the upper right corner of individual graphs.

To determine the predictive value of the examined DNA damage repair and tolerance factors with regard to CDDP response, we performed correlation of their individual as well as of cumulative pathway basal mRNA expression with the IC_50_ values of continuous 48 h CDDP exposure. Within the NER factors, we revealed a statistically significant correlation of IC_50_ only with the *XPA* expression level (Figure 7A; r^2^ = 0.818, *p* = 0.0052 and Supplementary Figure S2A–C). In case of HR and TLS, such correlation was found only for *RAD51C* (Figure 7B; r^2^ = 0.600, *p* = 0.0407 and Supplementary Figure S2D,E) and *POLH* (Figure 7C; r^2^ = 0.672, *p* = 0.0241 and Supplementary Figure S2F,G) mRNA levels. Although not with NER (Figure 7D; r^2^ = 0.432, *p* = 0.109), IC_50_ values also significantly correlated with the cumulative basal expression of the HR (Figure 7E; r^2^ = 0.680, *p* = 0.0225) and TLS (Figure 7F; r^2^ = 0.729; *p* = 0.0144) pathways.

#### 2.3.5. Effect of CDDP on Relative mRNA Expression of the NER, HR and TLS Factors

**Figure 8:**
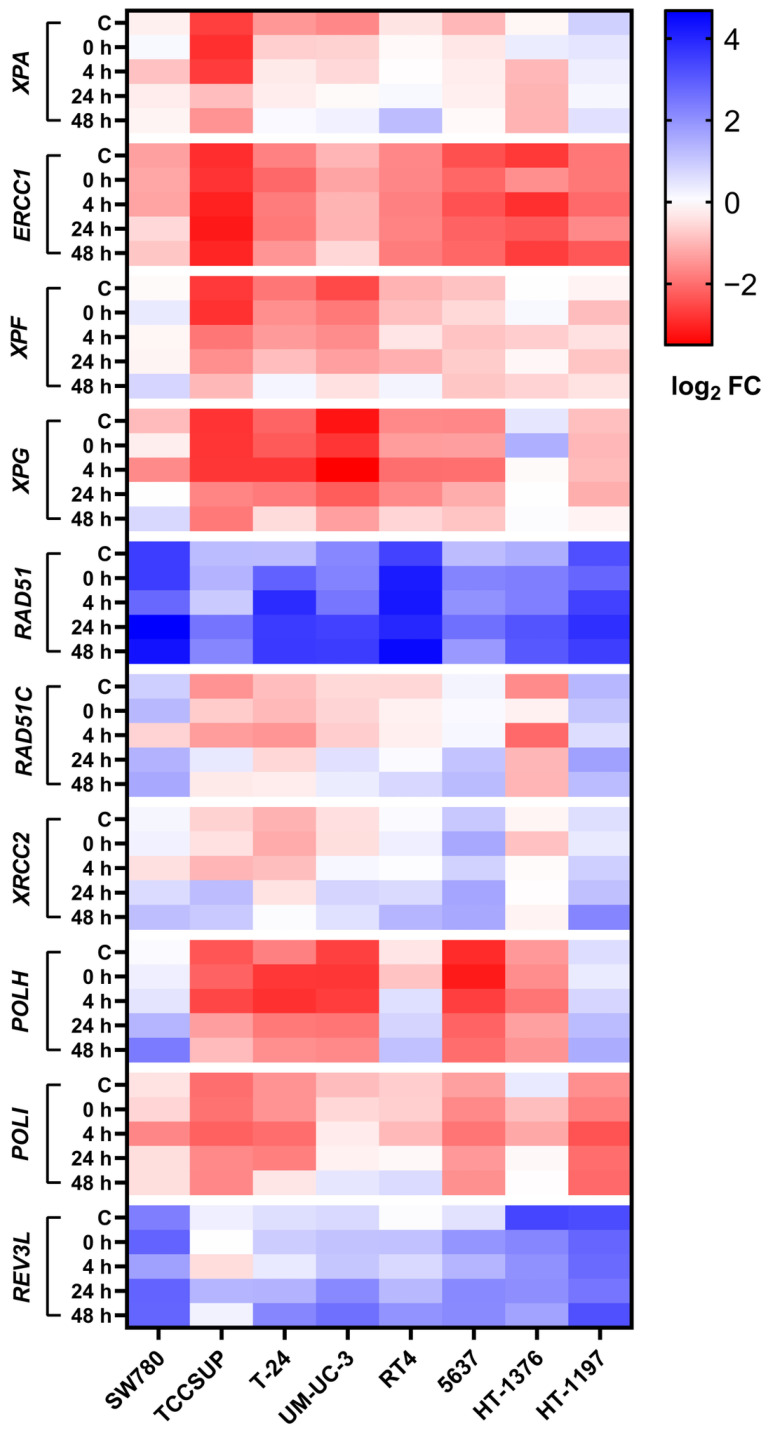
Changes in relative mRNA expression of the *XPA*, *ERCC1*, *XPF*, *XPG*, *RAD51*, *RAD51C*, *XRCC2*, *POLH*, *POLI* and *REV3L* genes after 2 h CDDP exposure at 0, 4, 24 and 48 h. The mRNA expression values of the respective genes are listed as the mean fold change of the transcript concentration (log_2_ FC) from the three biological and three technical replicates compared to BEC. Red colour indicates relative expression lower than BEC, white colour represents expression similar to BEC, and blue represents relative expression higher than BEC. C means basal expression.

To better understand DNA repair data obtained by the SO-modified comet assay, we measured relative mRNA expression of the NER, HR and TLS factors after 2 h CDDP treatment at 0, 4, 24 and 48 h. The obtained data are shown in Figure 8 and Table 2. Except *ERCC1*, all examined DNA damage repair and tolerance factors underwent significant expression change after CDDP exposure at least in some UCC line, with 12.3% and 87.7% of all changes being represented by decreased and increased expression, respectively (Table 2). Interestingly, CDDP-induced decreased expression was mainly observed for the *POLI* gene (5 in 9 events). In contrast, CDDP-induced increased expression was almost equally distributed among all the examined genes. Generally, NER, HR and TLS pathways did not differ significantly in terms of frequency of CDDP-induced expression change of their individual components. As in case of viability and DNA repair kinetics, the UCC lines differ in gene expression change of the DNA damage repair and tolerance factors after CDDP exposure, with TCCSUP and 5637 being highly responsive and HT-1197 and HT-1376 slightly responsive.

## 3. Discussion

CDDP was introduced into clinical practice in the late 1970s/early 1980s after it was approved by the US Food and Drug Administration for the treatment of testicular and ovarian tumours [33]. Since all the UCC lines (with exception of UM-UC-3) studied herein were established before CDDP became routinely used for the treatment of BC patients, we cannot retrospectively assess its effect on the treatment response/outcome of the donors [34,35,36,37,38,39,40].

In the present study, we examined viability of a panel of the UCC lines and BEC after CDDP treatment. As obvious, the UCC lines differ to some extent in viability after CDDP treatment. We suggest that this difference might, at least in part, be caused by mutations in the *TP53* gene, which could lead to delayed or aberrant response of the UCC lines to the drug [41,42]. Mutations in exon 5 of this gene (case of the T24 cell line) can lead to expression of a shortened version of the p53 protein with a shorter half-life and an altered conformation, resulting in affected nuclear activity [43]. The functional impact of the mutation in exon 10 (case of TCCSUP) consists in the cytoplasmic retention of the p53 protein and the loss of its transactivation function [44]. A missense transition of *TP53* at codon 250 in the HT-1376 cell line is associated with the loss of p53 DNA-binding function [45,46]. HT-1197 has an unusual point mutation in codon 365, which encodes the C-terminal part of the domain responsible for p53 tetramerization. This mutation likely results in expression of p53 with altered conformation and oligomerization ability [47]. UM-UC-3 and 5637 have a mutation in the fourth and eighth exon of the *TP53* gene, respectively, but the functional consequence of these mutations is not sufficiently elucidated yet [42,48]. Based on these facts, we propose that, in order to better predict CDDP treatment outcome in BC patients, more detailed analysis of p53 mutations and their functional consequences is crucially required and should routinely be provided. 

Another factor that can affect CDDP response is the proliferation rate of the exposed cells. Most cytostatic drugs, including CDDP, act mainly during the S cell cycle phase. As a consequence, rapidly proliferating cells are generally more sensitive to cytotoxic agents than slowly proliferating ones [49,50,51,52]. Based on data obtained herein, we consider the HT-1197, RT4 and HT-1376 cell lines to be the most resistant to CDDP, as these three cell lines showed the highest IC_50_ values after continuous 48 h CDDP exposure. Importantly, these three UCC lines have previously been reported to have a slow proliferation rate [53,54,55,56]. Xylinas et al. [57] determined viability of the 35 UCC lines after 48 h CDDP exposure and, based on the data obtained, divided these cell lines into three categories: extremely sensitive, intermediate and extremely resistant. Their panel contained all the UCC lines used herein, with none of them being categorised as extremely sensitive. In line with our findings, they placed HT-1197 and RT4 in the category of extremely resistant cell lines. Other UCC lines used in the present study were categorized by them as intermediately resistant, with a slight difference in order from the most sensitive to the most resistant one. We assume that negligible difference between their and our CDDP viability data is likely caused by different cell cultivation conditions and/or by the solvent used to dissolve the drug (DMSO in their case), with the latter factor being indeed shown to have an impact on cellular toxicity by affecting mitochondrial functions [58].

Next, we examined induction and repair of DNA damage after CDDP exposure in the UCC lines. In parallel, we monitored changes in the relative expression of the selected NER, HR and TLS genes after CDDP exposure. Obviously, the UCC lines differed in kinetics of DNA damage induction and repair, pointing to distinct mechanisms contributing to overall CDDP response. Based on our data, we suggest that the UCC lines significantly differ in “on-target” mechanisms of CDDP response and possibly share a certain “pre-target” mechanism, through which they may be able to reduce the amount of CDDP capable of attacking DNA. Observed difference in “on-target” mechanisms very likely involves DNA damage repair and tolerance mechanisms, as evidenced by data on CDDP-induced DNA damage removal and expression change of the NER, HR and TLS factors, although there is not a direct correlation between the DNA repair efficiency and the level of expression of these factors. We propose that mechanisms ensuring survival after CDDP exposure in the UCC lines apparently involve efficient DNA damage repair and signalling, absent or aberrant/deregulated apoptosis, and DNA damage tolerance. On the other hand, absent and/or aberrant DNA repair, the wildtype apoptotic response and low level of DNA damage tolerance are the main causes of cell death after CDDP exposure in these cell lines. Therefore, to precisely predict CDDP response in BC, detailed insights into mechanisms of DNA damage signalling, repair, tolerance and apoptosis are crucially required.

To determine predictive value of the examined NER, HR and TLS genes with respect to CDDP response in BC, we correlated their relative basal along with the cumulative pathway expression with IC_50_ values in the UCC lines after CDDP exposure. In these analyses, however, the SW780 cell line was omitted, as this cell line displayed a very high level of CDDP sensitivity compared to other UCC lines, despite high levels of expression of some DNA damage repair and tolerance factors. SW780 was originally isolated from patient suffering from grade I NMIBC. Although diagnosed relatively early, the disease progressed very quickly in this patient, and eight months after the initial diagnosis, tumour tissue was also found in the vaginal wall. Two months later, metastatic disease was confirmed, and five months later, the patient died. Before the surgical removal of the tumour, the patient was administered the antineoplastic therapeutic thiotepa, after which the patient’s status did not improve [34]. We suggest that the SW780 cell line very likely contains a minor subpopulation of aggressive cells resistant to CDDP and that this cell line rather represents a model of early metastatic BC than that of NMIBC.

We found a positive correlation of IC_50_ values with the *XPA* mRNA expression level, suggesting that the *XPA* expression level could represent a potential biomarker of CDDP response in BC. In line with this assumption, we have recently showed [59] that the testicular germ cell tumour (TGCT) patients with low XPA expression have better OS than patients with high expression of this protein. In the same study, the combined NER pathway (XPA + ERCC1 + XPF) expression directly correlated with OS, although ERCC1 and XPF on their own did not reach statistical significance. *XPA* expression was also evaluated in colorectal cancer patients, where, in contrast to our TGCT data, high XPA protein expression predicted longer OS [60]. Predictive value of *XPA*, *ERCC1* and *XPF* expression was also evaluated in head and neck squamous cell carcinoma (SCC) patients [61], but no correlation was found between the expression of these proteins and OS, when the entire cohort of patients was analysed. However, subgroup analysis revealed that high *ERCC1* expression was associated with significantly lower OS in oral SCC patients. On the other hand, high *XPA* expression was associated with increased OS in patients with oropharyngeal SCC. *XPF* expression was not associated with OS in any of the subgroups. The abovementioned findings strongly suggest that tissue specificity plays an important role in association between CDDP response and DNA repair capacity. Within the UCC lines, we did not notice any relationship between *ERCC1* mRNA expression and IC_50_ values. However, a significantly higher expression of this gene in BEC compared to the UCC lines may suggest that reduction of *ERCC1* expression could potentially be associated with the process of malignant transformation. It has been shown that *ERCC1* expression was significantly associated with longer survival in the group of BC patients without adjuvant chemotherapy, while ERCC1 positivity was associated with shorter survival in patients receiving adjuvant chemotherapy [62], indicating that administration of adjuvant chemotherapy has a clinical significance in patients with ERCC1 negative tumours. Similarly, ERCC1-positive patients indicated for adjuvant oxaliplatin-based chemotherapy had a shorter median survival compared to ERCC1-negative patients [63]. Moreover, an association between ERCC1 expression and OS was reported in BC, but only for patients who did not undergo CDDP-based adjuvant chemotherapy [64]. The same study also associated *ERCC1* mRNA expression with CDDP sensitivity in the 25 UCC lines and, similarly to our findings, no correlation between *ERCC1* mRNA levels and sensitivity to CDDP was observed. In addition, Hemdan et al. [65] found that *ERCC1* expression has a prognostic value only in patients who underwent RC without NAC and that NAC is beneficial for patients not expressing ERCC1 in tumours. Finally, meta-analysis including 1475 patients with advanced BC concluded that ERCC1 positivity may be a prognostic indicator of worse survival outcome in patients with advanced BC [66]. We suggest that, although prognostic value of *ERCC1* expression strongly depends on treatment regimen and disease characteristics in BC, it still represents a valuable factor in designing of prognosis scoring algorithm/cancer treatment algorithm.

Within HR, we disclosed a positive correlation of IC_50_ values with the level of *RAD51C* mRNA expression and with the cumulative expression of the HR pathway. The chromosomal region, in which the *RAD51C* gene is located, has been found to be often amplified in sporadic breast cancers. This amplification is associated with overexpression of *RAD51C* and several other genes in a significant proportion of the primary tumours [67,68]. In addition, *RAD51C* methylation appears to be a positive predictive biomarker of PARP inhibitor response in ovarian carcinomas, where loss of methylation even in one copy of the gene is sufficient to confer resistance to this inhibitor [69]. In ovarian cancer, *XRCC2* expression also directly associates with OS and PFS [70]. *XRCC2* expression was also analysed in silico using multiple data sets in relation to the prognosis of glioma patients. In contrast to ovarian cancer, *XRCC2* expression inversely correlates with glioma patient prognosis [71]. In case of *RAD51*, its low expression significantly correlates with better OS in neuroblastoma patients [72]. Interestingly, BEC expressed the lowest mRNA level of this crucial HR factor, although difference from other UCC lines was not statistically significant. Nevertheless, a low expression of *RAD51* in BEC compared to the UCC lines may suggest that an increase of its expression could potentially be associated with the process of malignant transformation in BC.

Cumulative mRNA expression of the TLS pathway after CDDP exposure also showed positive correlation with the IC_50_ values. At the level of individual TLS factors, only correlation of the *POLH* mRNA levels with IC_50_ was statistically significant. Previously, it has been found that loss of *POLH* significantly attenuates resistance to CDDP in both CDDP-sensitive and -resistant lung cancer cell lines [73]. The study also reported association of the *POLH* mRNA expression levels with the degree of CDDP resistance in a panel of the UCC lines that were identical to ours. In patients with NSCLC, head and neck SCC, and metastatic gastric cancer treated with CDDP-based chemotherapy, *POLH* expression predicts OS [74,75,76]. Increased expression of *POLI* positively correlates with the degree of malignancy in tumour samples from BC patients [77]. Knockdown of *REV3L* mRNA, in turn, mediates hypersensitivity to CDDP in head and neck SCC cells [78]. The recent study of Sakurai et al. [79] showed that inactivation of *REV7* increases chemosensitivity and overcomes chemoresistance of TGCT cell lines. However, the role of TLS in chemoresistance to CDDP in BC is far from being explained, and its closer understanding could represent a benefit, especially in the form of new potential therapeutic targets.

The work currently ongoing in the laboratory tries to address clinical importance of the DNA damage repair and tolerance factors examined herein using a large cohort of BC patients. In addition, BEC and the MIBC UCC lines have recently be profiled for the whole genome mRNA and miRNA expression before and after CDDP treatment in order to identify factors that strongly associate with CDDP response. Those factors are subsequently to be examined for their expression in cohort of MIBC patients to address their predictive value in terms of outcome of CDDP-based chemotherapy. Hopefully, these studies potentially contribute to a deeper understanding of CDDP response in MIBC and disclose a role of DNA damage repair and tolerance mechanisms in the process.

## 4. Materials and Methods

### 4.1. Cell Lines

A panel of the eight UCC lines (TCP-1020) and BEC (PCS-420-010) were used. All were purchased from the American Type Culture Collection (ATCC, Manassas, VA, USA). All cell lines were cultured in a 5% CO_2_ atmosphere at 37 °C in appropriate culture media. The HT-1197, HT-1376, UM-UC-3 and TCCSUP cell lines were cultured in EMEM, 5637 and SW780 in RPMI-1640, and RT4 and T-24 in McCoy’s 5A medium (all media were from ATCC). All culture media for the UCC lines were supplemented with 10% fetal bovine serum (Gibco, Thermo Fisher Scientific, Horsham, UK) and 1% penicillin/streptomycin (10,000 U/mL/10,000 μg/mL; Gibco, Life Technologies, Grand Island, NY, USA). BEC were cultured in basal medium for bladder epithelial cells (PCS-420-032; from ATCC) enriched with components of the growth kit (PCS-420-042; from ATCC).

### 4.2. Cell Viability Assay

3-(4,5-dimethylthiazol-2-yl)-2,5-diphenyltetrazolium bromide (MTT; stock solution = 1 mg/mL; Sigma-Aldrich, St. Louis, MO, USA) was used in cell viability assay to determine the IC_50_ value of CDDP (a gift from the National Cancer Institute, Bratislava, Slovak Republic). The UCC lines were seeded in a 96-well plate at an initial number of 8 × 10^3^ cells/well in volume of 100 μL of the appropriate culture medium and left to adhere for 24 h under standard incubation conditions. After 24 h incubation, the cells were treated with CDDP in four different ways: (a) 2 h treatment (CDDP concentrations used: 0, 5, 10 and 20 μg/mL) followed by 1x phosphate-buffered saline (PBS; 137 mM NaCl; 2.7 mM KCl; 10 mM Na_2_HPO_4_, 1.8 mM KH_2_PO_4_, pH 7.4) wash and 24 h post-incubation in fresh medium, (b) 2 h treatment (CDDP concentrations as in a)) followed by 1× PBS wash and 48 h post-incubation in fresh medium, (c) 24 h treatment (CDDP concentrations used: 0, 1.25, 2.5, 5, 7.5, 10, 12.5, 15, 20 and 40 μg/mL), and (d) 48 h treatment (CDDP concentrations as in (c)). Prior to adding MTT, the cells were 1x washed with PBS. The cells were then incubated at 37 °C for 4 h in a mixture consisting of culture medium (100 μL) and MTT (50 μL). After 4 h incubation, the MTT solution was aspirated and 50 μL of dimethylsulfoxide (DMSO; Serva, Heidelberg, Germany) were added to dissolve MTT formazan during 15 min of shaking in the dark. The absorbance at 540 nm (reference wavelength: 690 nm) was measured on an xMark ™ Microplate Spectrophotometer (Bio-Rad Laboratories, Hercules, CA, USA). In each experiment, the MTT test results and the inhibitory effect of CDDP on cell growth were converted to a percentage of viable cells compared to untreated control. The MTT assay was repeated in three biological and eight technical replicates for each cell line.

### 4.3. Comet Assay

Cells were seeded in 24-well plates at a density of 1 − 1.5 × 10^5^ cells/well in the appropriate culture medium and incubated at 37 °C for 24 h. Afterwards, the cells were treated with 5, 10 and 20 μg/mL CDDP (one aliquot was left untreated) for 2 h. After the treatment, the cells were 1× washed with PBS and then resupended in fresh medium to allow them to recover for 4, 24 and 48 h (one aliquot was processed immediately after the treatment with no recovery). To monitor CDDP-induced DNA damage, styrene oxide (SO; Thermo Fisher Scientific; Waltham, MA, USA; dissolved in DMSO) in a final concentration of 900 μM was added to samples 30 min prior to conducting the comet assay. Samples for each time interval consisted of untreated cells, cells treated only with SO, cells treated only with CDDP and cells treated with the combination of SO and CDDP.

The cells were then subjected to the comet assay under alkaline conditions with modifications according to Ostling and Johanson [80] and Singh et al. [81]. After trypsinization, the cells were transferred to microtubes at the indicated time intervals and pelleted by centrifugation. The supernatant was removed and the cell pellets were resuspended in low melting point agarose (Invitrogen, Life Technologies, Carlsbad, CA, USA) pre-heated to 37 °C. A total of 50 μL of cell suspension were applied to slides, and once a thin agar film was formed, the samples were incubated in a lysis solution (2.5 M NaCl, 10 mM Tris-HCl, 100 mM Na_2_EDTA, 1% TritonX, pH 10) at 4 °C for 1 h. After lysis, the slides were incubated in chilled electrophoretic buffer (1 mM Na_2_EDTA, 0.3 M NaOH, pH 13) for 40 min and then electrophoresed at 25 V and 300 mA for 30 min. Upon completion, the samples were washed twice with a cold neutralization solution (0.4 M Tris-HCl, pH 7.5). Finally, the samples were washed with distilled water and then air-dried at room temperature. Samples were stained with ethidium bromide (30 μg/mL in distilled water; Sigma-Aldrich, Saint Louis, MO, USA) just before being analysed using a Carl Zeiss AxioImager.Z2 fluorescence microscope and Metafer 5 Slide Scanning Platform software (Metasystems, Altlussheim, Germany). The percentage of DNA in the comet tail was chosen as a parameter for expression of the level of DNA damage. The comet assay was performed in three biological replicates, in which each sample was performed in two technical replicates.

### 4.4. Quantification of Relative mRNA Expression by Real-Time Polymerase Chain Reaction (RT-qPCR)

TRI Reagent (Life Technologies, Carlsbad, CA, USA) was used to isolate the total RNA from the UCC lines. The concentration and purity of the isolated RNA was determined with a NanoDrop ND-100 spectrophotometer (Thermo Fisher Scientific, Waltham, MA, USA). The expression of the examined genes (*XPA*, *ERCC1*, *XPF*, *XPG*, *RAD51*, *RAD51C*, *XRCC2*, *POLH*, *POLI* and *REV3L*) was determined by RT-qPCR. An input of 1.5 μg of the total RNA and RevertAid First strand cDNA Synthesis Kit (Thermo Fisher Scientific, Waltham, MA, USA) was used to transcribe RNA into cDNA. For cDNA synthesis in a reaction of a final volume of 20 μL, 1500 ng of total RNA, 4 μL of 5× Reaction buffer, 1 μM of random hexamer primer, 0.1 mM of dNTP mixture and 100 units of RevertAid Reverse Transcriptase, a recombinant M-MuLV RT, were incubated at 42 °C for 1 h followed by enzyme inactivation at 95 °C for 5 min. qPCR was performed using SYBR Premix Ex Taq II (Tli RNaseH Plus, Takara Bio Inc., Kusatsu, Japan) with specific forward and reverse primers listed in Table S1 in the Supplementary Materials. Agilent thermocycler, ARIA Real-Time PCR System was used for qPCR reaction with following amplification program: 95 °C for 5 min, 40 cycles of 95 °C for 20 s and 60 °C for 50 s, followed by a melting curve cycle. Amplification of each gene was performed in three biological and three technical replicates. Phosphoglycerate kinase (*PGK1*) and β-actin (*ACTB*) were used as control reference genes. To evaluate the effect of CDDP on expression change of examined selected DNA repair genes, as well as of cumulative NER, HR and TLS pathways, the UCC lines were identically CDDP treated as in the comet assay experiments. Cumulative expression represents sum of relative expression (2^−ΔCt)^ of the analysed genes (e.g., *XPA* + *ERCC1* + *XPF* + *XPG*, *RAD51* + *RAD51C* + *XRCC2* and *REV3L* + *POLH* + *POLI* for NER, HR and TLS, respectively).

### 4.5. Statistical Analysis

SigmaPlot 12.5 and Prism GraphPad 8.4.3 software were used for statistical analysis. The IC_50_ value for the cell lines was determined by non-linear regression. Analysis of the gene expression changes was evaluated from the ΔCt values (threshold cycle value) normalized to the geometric mean of the reference genes. This means that ΔCt = Ct (gene of interest) −ΔCt (geometric mean Ct (*PGK1* and *ACTB*)). The relative quantification of the basal mRNA expression of the examined genes was evaluated by the 2^−ΔCt^ method and is reported as the mean ± SD. The heatmap showing change in the gene expression after 2 h CDDP treatment and subsequent post-cultivation in fresh medium was evaluated by the method 2^−ΔΔCt^, i.e., ΔΔCt = ΔCt (urothelial cancer cell line) −ΔCt (BEC). Heatmap values are reported as log_2_ of the average transcript concentration (FC) ratio. The normality of the data distribution was assessed by the Shapiro–Wilk test. Homogeneity of variance was assessed by Leven’s test. In the case of parametric data distribution, one-way analysis of variance (ANOVA) with Tukey post-hoc test for all-pairwise multiple comparison and a Bonferoni post-hoc test for multiple comparison against control sample were used. For parametric distribution of data showing a heteroscedasticity, a one-way ANOVA with Tamhane T2 post-hoc test was applied. For non-parametric data distribution, Kruskall–Wallis ANOVA with Dun’s post-hoc test was used. Pearson correlation was performed to determine the association between the basal gene expression and IC_50_ value. IC_50_ values for individual cell lines were calculated using non-linear regression.

## 5. Conclusions

As CDDP-based chemotherapy is a standard of care in neoadjuvant setting, as well as first line therapy of advanced BC, in the present study we have examined CDDP response in a panel of the UCC lines. We monitored viability after CDDP treatment and kinetics of induction and repair of CDDP-induced DNA damage in these cell lines. Here, we show that response of UCC to CDDP is strongly dependent on treatment conditions and that these cell lines display a complex pattern of response to CDDP. Furthermore, we demonstrate that repair of CDDP-induced DNA lesions correlates to some extend with CDDP sensitivity. We also monitored expression of the key genes involved in selected DNA damage repair and tolerance mechanisms and show that it differs in the UCC lines and positively correlates with viability after CDDP exposure. Our data indicate that CDDP response in BC is dependent on DNA damage repair and tolerance factors, which may, therefore, represent valuable therapeutic targets in this malignancy. However, further work is still needed to fully establish the role of examined DNA damage repair and tolerance factors in CDDP response in BC.

## Figures and Tables

**Table 1:** IC_50_ values for the UCC lines and BEC for all CDDP treatment conditions. CDDP, cisplatin; CI, confidence interval; NA, not applicable.

UCC Line/BEC	IC_50_ (μg/mL CDDP) (95% CI)			
	2 h Treatment with 24 h Post-Incubation	2 h Treatment with 48 h Post-Incubation	24 h Continuous Treatment	48 h Continuous Treatment
BEC	NA	23.72 (20.49–27.65)	7.78 (7.40–8.16)	2.62 (2.44–2.80)
HT-1197	20.89 (19.64–22.52)	14.64 (13.95–15.37)	6.85 (6.33–7.39)	5.58 (5.21–5.95)
HT-1376	15.57 (14.70–16.58)	11.27 (10.75–11.82)	11.65 (11.05–12.29)	3.54 (3.32–3.76)
TCCSUP	22.47 (20.04–26.07)	11.26 (10.70–11.86)	12.25 (11.26–13.36)	1.27 (1.20–1.33)
5637	20.86 (19.89–22.01)	16.65 (15.78–17.66)	6.09 (5.82–6.36)	2.87 (2.74–3.01)
RT4	16.14 (14.57–18.24)	11.40 (9.68–13.60)	5.49 (5.22–5.76)	3.74 (3.58–3.90)
SW780	10.99 (10.46–11.56)	6.34 (6.07–6.61)	3.93 (3.75–4.11)	2.39 (2.24–2.54)
T-24	25.55 (22.12–31.07)	4.79 (4.46–5.10)	12.91 (11.96–13.99)	2.17 (2.02–2.32)
UM-UC-3	14.94 (14.06–15.97)	4.98 (4.82–5.14)	6.33 (5.90–6.78)	3.48 (2.97–4.00)

**Table 2:** Relative expression of the NER, HR and TLS factors after 2 h CDDP treatment at 0, 4, 24 and 48 h. NS, not significant. Blue and red colour refers to an increased and decreased expression due to CDDP treatment, respectively. Statistical significance: * *p* < 0.05; ** *p* < 0.01; *** *p* < 0.001.

UCC Line		*XPA*	*ERCC1*	*XPF*	*XPG*	*RAD51*	*RAD51C*	*XRCC2*	*POLH*	*POLI*	*REV3L*
		mRNA FC (min–max)									
SW780	0 h	NS	NS	NS	NS	NS	NS	NS	NS	NS	NS
	4 h	NS	NS	NS	*	NS	***	NS	NS	***	NS
	24 h	NS	*	NS	**	NS	*	NS	**	NS	NS
	48 h	NS	NS	NS	***	NS	**	***	***	NS	NS
TCCSUP	0 h	NS	NS	NS	NS	NS	NS	NS	NS	NS	NS
	4 h	NS	NS	NS	NS	NS	NS	NS	NS	NS	NS
	24 h	**	NS	NS	*	**	***	***	*	NS	NS
	48 h	*	NS	*	*	*	***	***	**	NS	NS
T-24	0 h	NS	NS	NS	NS	NS	NS	NS	NS	NS	NS
	4 h	NS	NS	NS	NS	NS	NS	NS	NS	NS	NS
	24 h	NS	NS	**	NS	NS	NS	NS	NS	NS	NS
	48 h	*	NS	***	**	NS	NS	**	NS	**	**
UM-UC-3	0 h	NS	NS	NS	NS	NS	NS	NS	NS	NS	NS
	4 h	NS	NS	NS	NS	NS	NS	NS	NS	*	NS
	24 h	**	NS	NS	**	NS	NS	***	NS	**	*
	48 h	***	NS	***	***	NS	NS	**	NS	***	**
RT4	0 h	NS	NS	NS	NS	NS	NS	NS	NS	NS	NS
	4 h	NS	NS	NS	NS	NS	NS	NS	NS	NS	NS
	24 h	NS	NS	NS	NS	NS	NS	NS	NS	NS	*
	48 h	***	NS	NS	*	NS	**	***	NS	***	**
5637	0 h	NS	NS	NS	NS	NS	NS	NS	NS	NS	*
	4 h	*	NS	NS	NS	NS	NS	NS	NS	*	NS
	24 h	*	NS	NS	NS	**	*	**	NS	NS	**
	48 h	*	NS	NS	*	NS	**	*	*	NS	*
HT-1376	0 h	NS	NS	NS	NS	NS	NS	NS	NS	*	NS
	4 h	NS	NS	NS	NS	NS	NS	NS	NS	**	NS
	24 h	NS	NS	NS	NS	*	NS	NS	NS	NS	NS
	48 h	NS	NS	NS	NS	*	NS	NS	NS	NS	NS
HT-1197	0 h	NS	NS	NS	NS	NS	NS	NS	NS	NS	NS
	4 h	NS	NS	NS	NS	NS	NS	NS	NS	NS	NS
	24 h	NS	NS	NS	NS	NS	NS	NS	***	NS	*
	48 h	NS	NS	**	NS	NS	NS	NS	***	NS	NS

## Data Availability

Rough data available on request.

## Supplementary Materials

The following supporting information can be downloaded at: https://www.mdpi.com/article/10.3390/ijms232012488/s1, Figure S1: DNA damage induction and repair in the UCC lines and BEC after CDDP treatment. BEC (A), HT-1197 (B), HT-1376 (C), TCCSUP (D), RT4 (E), SW780 (F), T-24 (G), UM-UC-3 (H) a 5637 (I). Blue columns represent CDDP treatment followed by SO addition, while the red ones depict CDDP treatment only. Black asterisk shows statistical significance against SO-treated control (0 μg/mL, blue columns), while white asterisk shows statistical significance against CDDP-treated control (0 μg/mL, red columns). * *p* < 0.05, ** *p* < 0.01 and *** *p* <0.001 (for details, see Material and Methods), Figure S2: Correlation of relative basal mRNA expression of the *ERCC1* (A), *XPF* (B), *XPG* (C), *RAD51* (D), *XRCC2* (E), *POLI* (F) and *REV3L* (G) genes with IC_50_ values of continuous 48 h CDDP treatment, Table S1: List of primers used for RT-qPCR analysis.
