# Supplementary material for: Response of the Urothelial Carcinoma Cell Lines to Cisplatin
Source: Int J Mol Sci. 2022 Oct 18;23(20):12488. doi: 10.3390/ijms232012488 (PMC9604399; doi:10.3390/ijms232012488)
Supplement: Supplementary file 1 [file ijms-23-12488-s001.zip › ijms-1883068-supplementary.pdf]

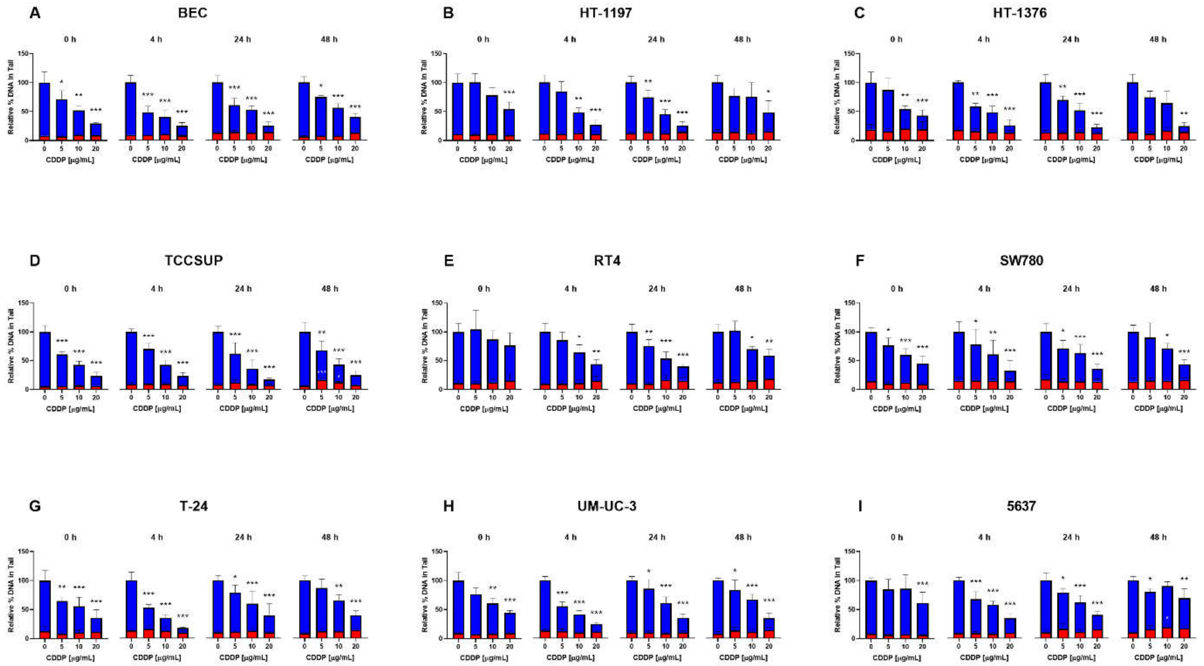

Figure S1. DNA damage induction and repair in the UCC lines and BEC after CDDP treatment. BEC (A), HT-1197 (B), HT-1376 (C), TCCSUP (D), RT4 (E), SW780 (F), T-24 (G), UM-UC-3 (H) a 5637 (I). Blue columns represent CDDP treatment followed by SO addition, while the red ones depict CDDP treatment only. Black asterisk shows statistical significance against SO-treated control (0 µg/mL, blue columns), while white asterisk shows statistical significance against CDDP-treated control (0 µg/mL, red columns). *$p < 0.05$, **$p < 0.01$ and ***$p < 0.001$ (for details, see Material and Methods).

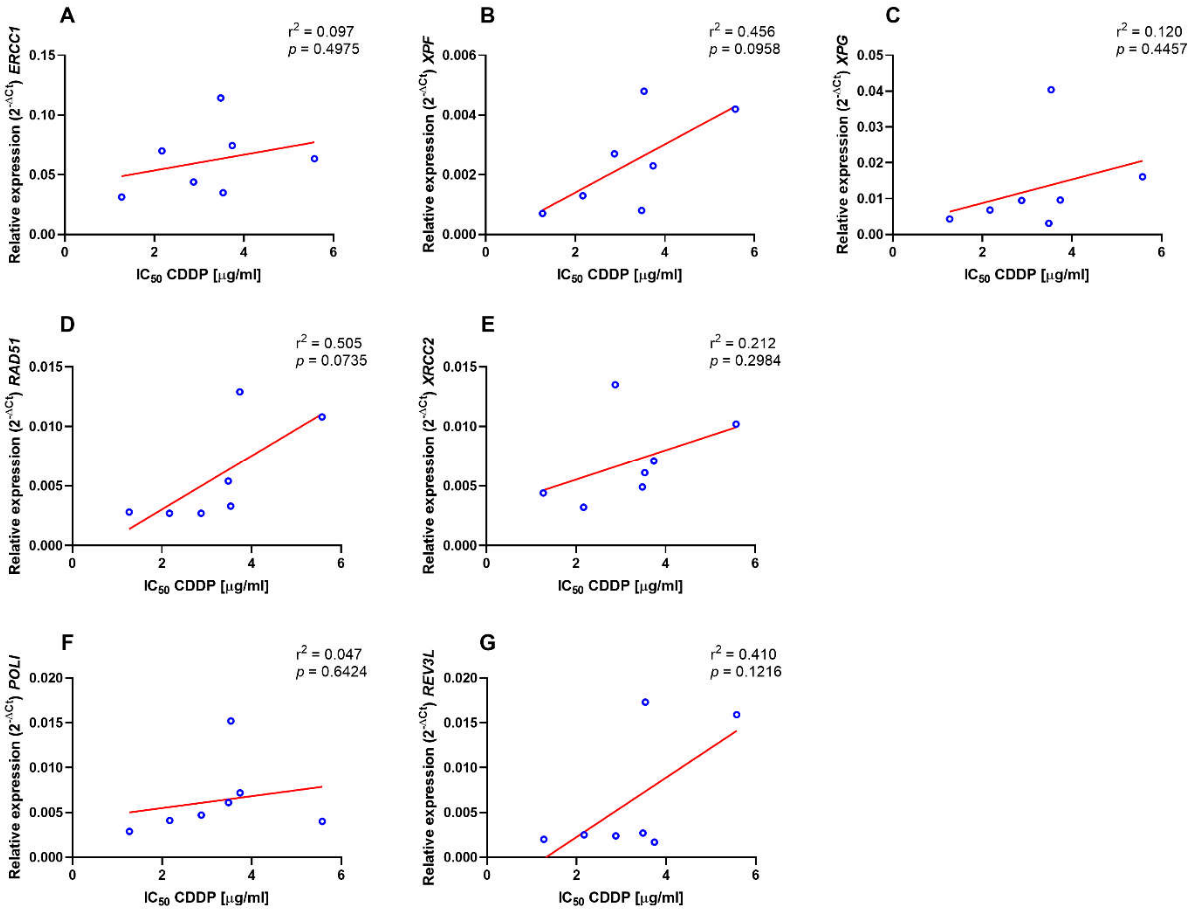

Figure S2. Correlation of relative basal mRNA expression of the *ERCC1* (A), *XPF* (B), *XPG* (C), *RAD51* (D), *XRCC2* (E), *POLI* (F) and *REV3L* (G) genes with IC_{50} values of continuous 48 h CDDP treatment.

Table S1. List of primers used for RT-qPCR analysis.

Gene	Forward (5′– 3′)	Reverse (5′– 3′)
XPA	TCACAATGGGGTGATATGAAACTC	CTGTCGGACTTCCTTTGCTTC
ERCC1	CTGCTTGTCCAGGTGGATG	GCTGGTTTCTGCTCATAGGC
XPF	AACCTTTGTTCGGCAGCTTG	GCGTTGTTCCTCAGTTGAACC
XPG	CAAAGGCCGTGGAACCAATG	TTCGCCTTGTTCTGAGGGAG
RAD51	AGAAGTGGAGCGTAAGCCAG	GAAATGGGTTGTGGGCCAAAG
RAD51C	AGTTGGGGACATGCTGCTAC	ATTCCTTCTGGCTGGGTGAC
XRCC2	GTTGGTGAATGGCGTTGGTG	TACCTTCAAGTCGGGCAAGG
POLH	TCGGGAACAGGTACAATGGTGG	ATAGCGGGTAAGGGCACAGC
POLI	CACCAAATGCTTCATCCAGAG	TCCTAGCTTCATAGTTGCAGG
REV3L	GGGTGCCAGATGACAAAATTGAG	AGAGGGGAAAACGCTGATGAAG
PGK	TGGAGCTCCTGGAAGGTAAAG	AAGTTGACTTAGGGGCTGTGC
ACTB	GCACTCTTCCAGCCTTCCTT	CGTACAGGTCTTTGCGGATG
